# Dataset for microstructure and mechanical properties of (CrCoNi)97Al1.5Ti1.5 medium entropy alloy twisted by free-end-torsion at room and cryogenic temperatures

**DOI:** 10.1016/j.dib.2020.106333

**Published:** 2020-09-23

**Authors:** Yinglong Zhao, Ning Guo, Shiteng Long, Xiaobin Huang, Bo Song, Jianjun Hu, Linjiang Chai, Shengfeng Guo

**Affiliations:** a School of Materials and Energy, Southwest University, Chongqing 400715, China; b College of Materials Science and Engineering, Chongqing University of Technology, Chongqing 400054, China

**Keywords:** CrCoNi, Medium entropy alloy, Torsion-test data, Gradient dislocation, Gradient materials

## Abstract

This data article presents the torsion parameters and the microstructural data of the (CrCoNi)97Al1.5Ti1.5 medium-entropy alloy (MEA). The data presented in this article are related to the research article entitled “Microstructure and mechanical properties of (CrCoNi)_97_Al_1.5_Ti_1.5_ medium entropy alloy twisted by free-end-torsion at room and cryogenic temperatures”, see Ref. [1]. This article can be used for data analysis and interpretation and their comparison with other data sets in the research articles. The microstructure and the element distributions of the as-swaged rods were obtained using a scanning electron microscope (SEM) equipped with electron channelling contrast imaging (ECCI), electron diffraction spectroscopy (EDS) and electron backscattered diffraction (EBSD) detectors. The phases of the MEA before and after torsion are determined by the X-ray diffractometer (XRD) techniques. Optical micrograph, inverse pole figure (IPF) map, grain boundary map and misorientation angle distribution and pole figure of the as-swaged sample were presented. I In order to provide data reference for future torsion experiments, this article draws schematic diagrams of the hot-swaged rod, dimensions of the torsion/tensile specimens, liquid nitrogen (@LN) environment torsion device and schematic representation for characterization locations of microstructure. Lastly, Kernel Average Misorientation (KAM) maps and misorientation angle distribution of various samples or different strained layers were used for comparative analysis.

## Specifications Table

**Table:** 

Subject	Materials Science
Specific subject area	High- and medium-entropy alloys (HEAs and MEAs)
Type of data	Figures (scanning electron microscopy; EBSD maps; X-ray patterns; and Schematic diagrams of torsion samples) and “txt” data.
How data were acquired	SEM: Zeiss Sigma HD; EBSD:Oxford AZtech Max2; XRD: MAXima XRD-6100, ShimadzuX-ray Diffractometer
Data format	Raw data (EDS images, EBSD images torsion schematic diagrams figures in *.PSD; XRD patterns in *.opj and; EBSD KAM data and Misorientation Angle distribution data in *.txt)
Parameters for data collection	Backscatter electron images were obtained using SEM with acceleration voltages of 20 kV. XRD patterns were obtained using X-ray diffractometer at a scanning step 0.02° from 20° to 100°.
Description of data collection	Metallographic samples were cut, embedded and prepared by grinding and polishing.
Data source location	School of Materials and Energy, Southwest University, Chongqing 400715, China
Data accessibility	Data are with the article (attached file)
Related research article	N. Guo, Y. Zhao, S. Long, B. Song, J. Hu, B. Gan, L. Chai, S. Guo, Microstructure and mechanical properties of (CrCoNi)_97_Al_1.5_Ti_1.5_ medium entropy alloy twisted by free-end-torsion at room and cryogenic temperatures, Mater. Sci. Eng. A. Volume 797, 21 October 2020, 140101.

## Value of the Data

• These data are useful for insight into the influence of torsion strain and temperature on the microstructure of the CrCoNi medium-entropy alloy and other similar alloys with low-stacking faults.
• These data can provide useful information for the structural design and controllable manufacturing of high-performance gradient materials, including methods and parameters.
• The electron backscattered diffraction (EBSD) results of the various twisted samples can be used to quantitatively analyze the strengening mechanisem of the materials with gradient dislocations.

## 1 Data Description

**Fig. 1:**
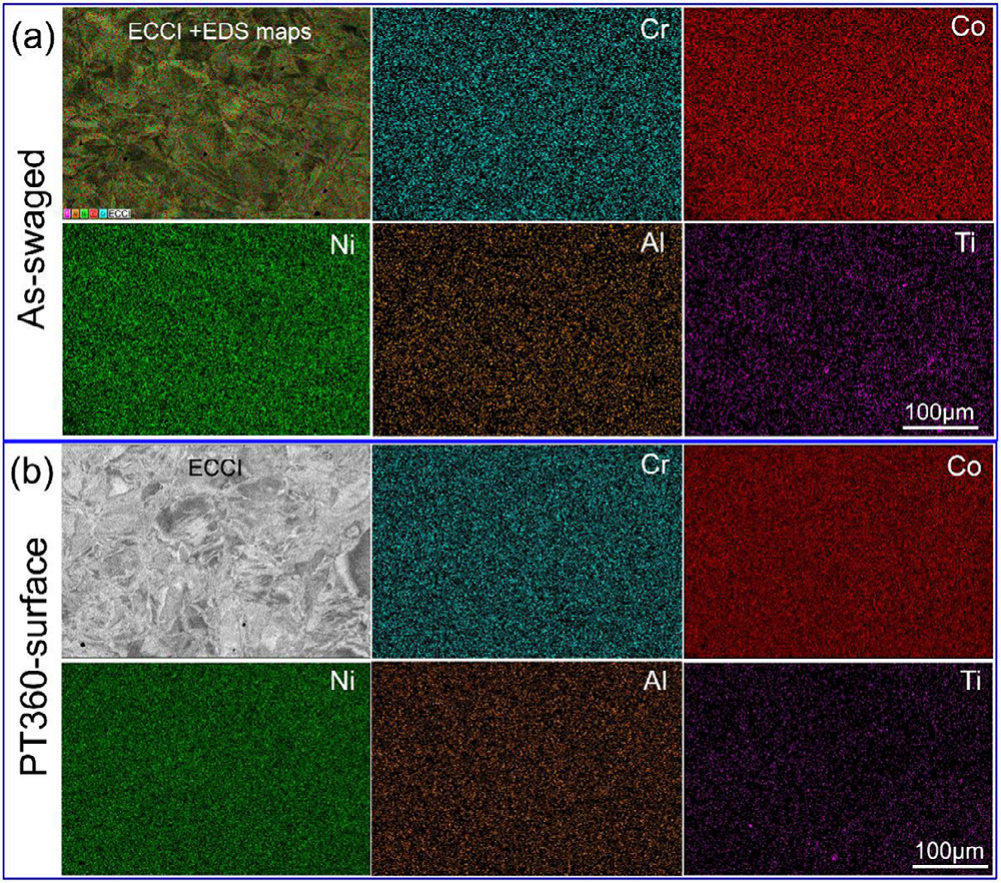
EDS maps showing element distributions of the as-swaged sample (a) and surface layer of PT360 sample (b).

**Fig. 2:**
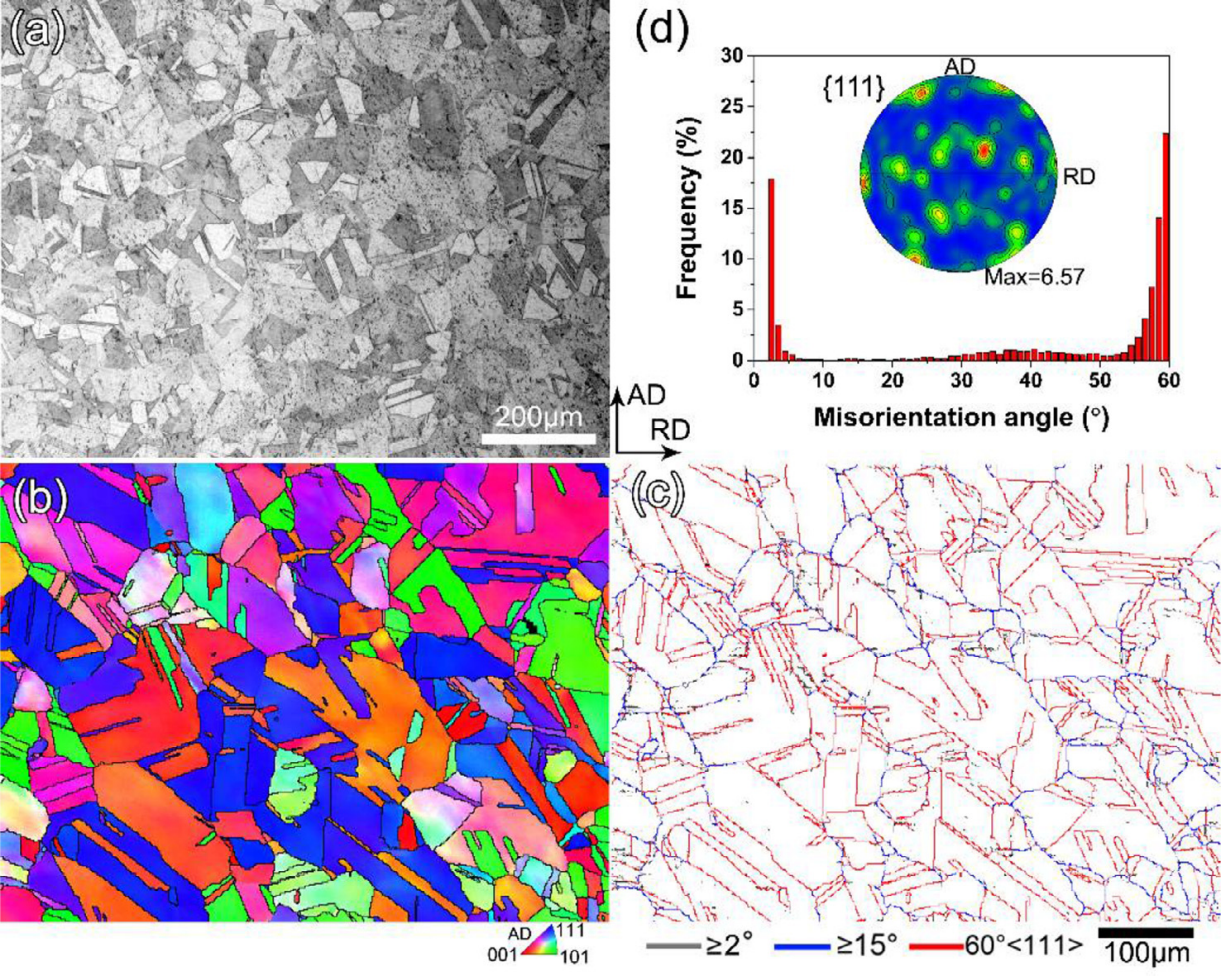
Optical micrograph (a), EBSD IPF map (b), grain boundary (GB) map (c) and misorientation angle distribution and {111} pole figure (d) of the as-swaged sample.

**Fig. 3:**
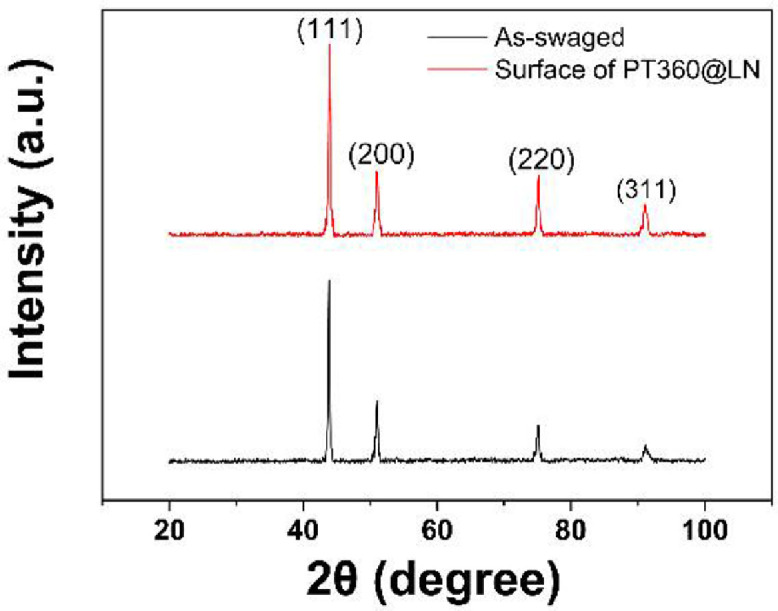
XRD patterns of As-swaged and PT360@LN samples. For the PT360@LN, the data was collected from the surface of the sample (multiple surface-layer pieces were spliced together, the area is about 64 cm^2^).

**Fig. 4:**
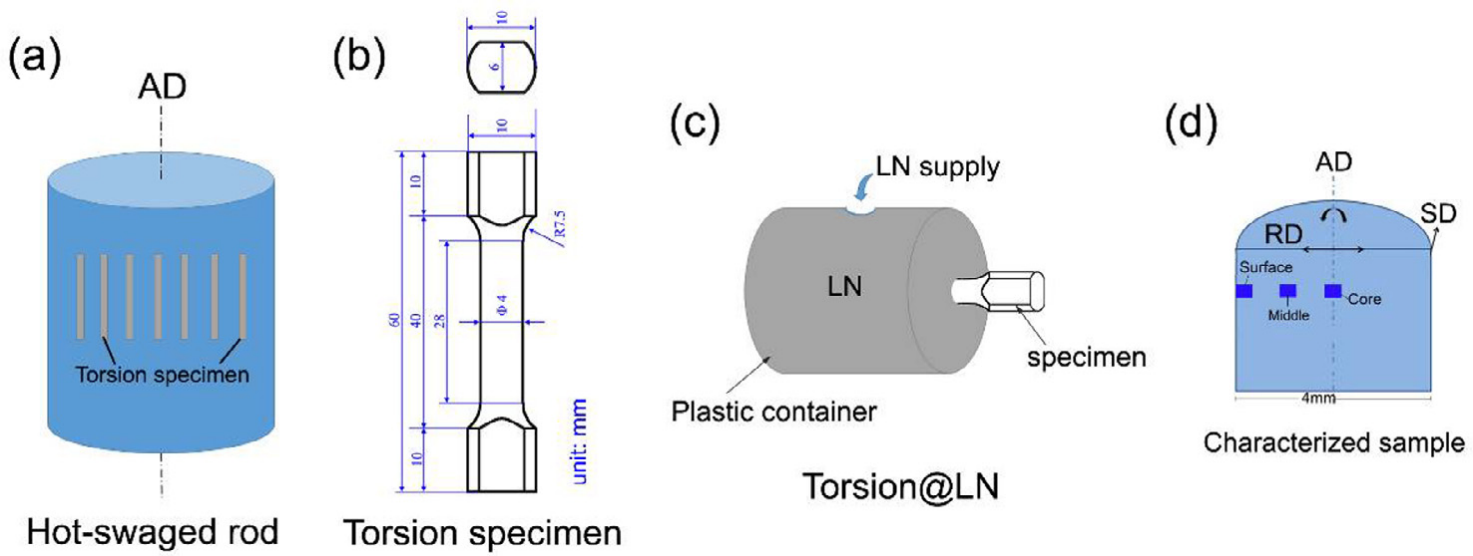
Schematic diagrams showing the hot-swaged rod (a), dimensions of the torsion/tensile specimens (b), liquid nitrogen (LN) environment torsion device (c) and schematic representation for characterization locations of microstructure (d). AD, SD and RD mean axial direction, shear direction and radial direction, respectably. The unit in (b) is mm.

The detailed torsion data and microstructure data are presented in this section. Element distributions of the as-swaged sample are shown in Fig. 1a, and b shows the element distributions in the surface layer of the sample after being twisted 360 ° at room temperature. Fig. 2 presents the optical micrograph and EBSD results of the as-swaged sample. Fig. 2b is the EBSD IPF map, and Fig. 2c is the grain boundary (GB) map with low-angle boundaries (LABs), high-angle boundaries (HABs) and twins highlighted in grey, blue and red, respectively. Fig. 2d shows misorientation angle distribution and {111} pole figure. XRD was used to identify the phase components of the as-swaged sample and twisted samples, as shown in Fig. 3. Free-end torsion experiments were carried out at room temperature and liquid nitrogen temperature, respectively. Fig. 4 shows how the twisted samples are cut from the swaged rod (Fig. 4a), the dimension of the twisted samples (Fig. 4b), the schematic diagram of the liquid nitrogen temperature twisting device (Fig. 4c), and microstructure characterization locations of the samples (Fig. 4d).

**Fig. 5:**
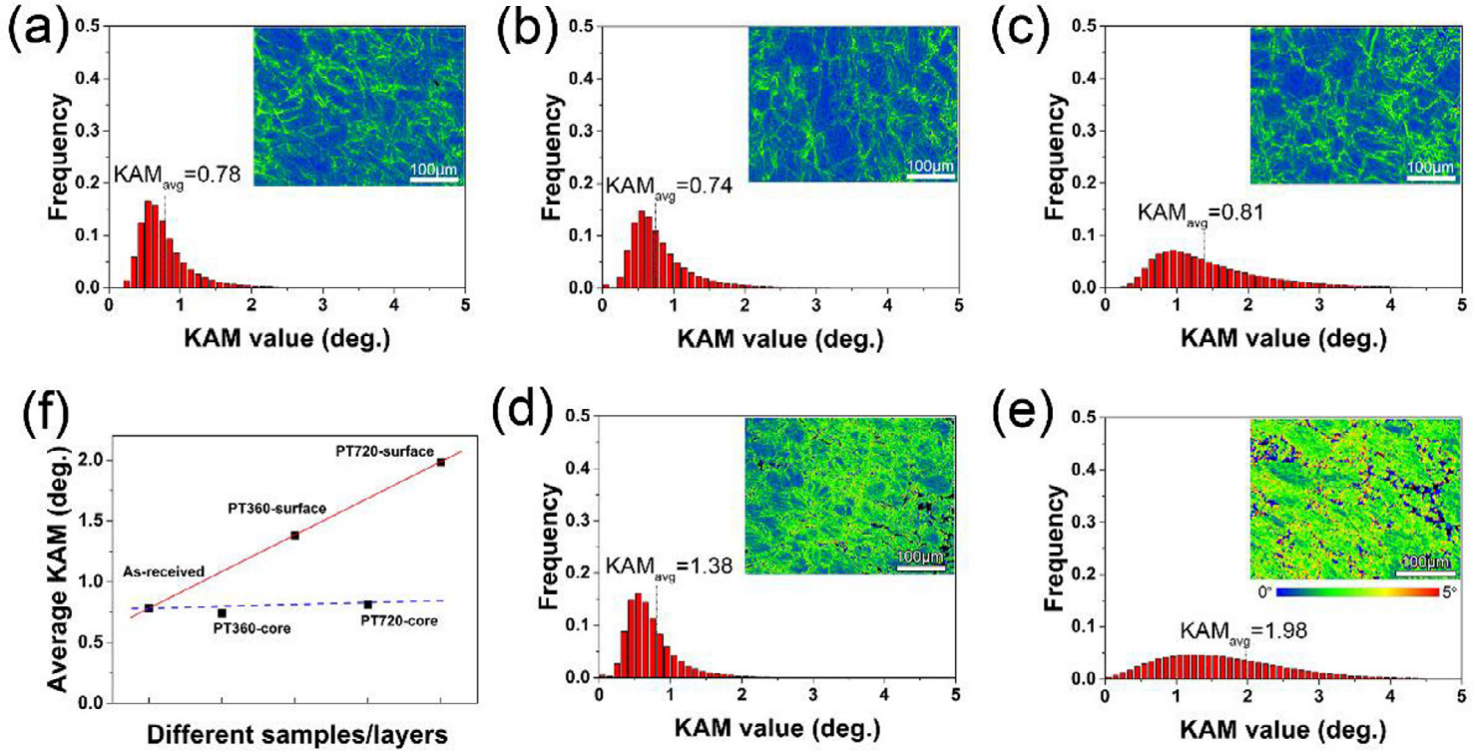
Kernel Average Misorientation (KAM) maps and distribution of various samples or different strained layers: (a) As-swaged; (b) core of PT360; (c) core of PT720; (d) surface of PT360; (e) surface of PT720; (f) average KAM evolution.

Kernel Average Misorientation (KAM) maps calculated from the EBSD data are shown in Fig. 5. KAM values are considered to be related to local misorientation (geometrically necessary dislocation “GND” density and distribution) inside grains [2,3]. The average KAM value of the untwisted sample, core of PT360 and core of PT720 are very small, and the high KAM values are mainly concentrated near the LABs rather than the grain interior (see Fig. 5a–c). It means that the dislocation density changes little and almost no plastic deformation occurs at the core region during twisting. The average KAM value of the PT360 and PT720 samples increases from 0.74 and 0.81 to 1.38, respectively, and more high KAM values are located in the grain interior (see Fig. 5d and e), indicating that plastic deformation occurs at the surface layer leading to an increase in GND density inside the grain.

**Fig. 6:**
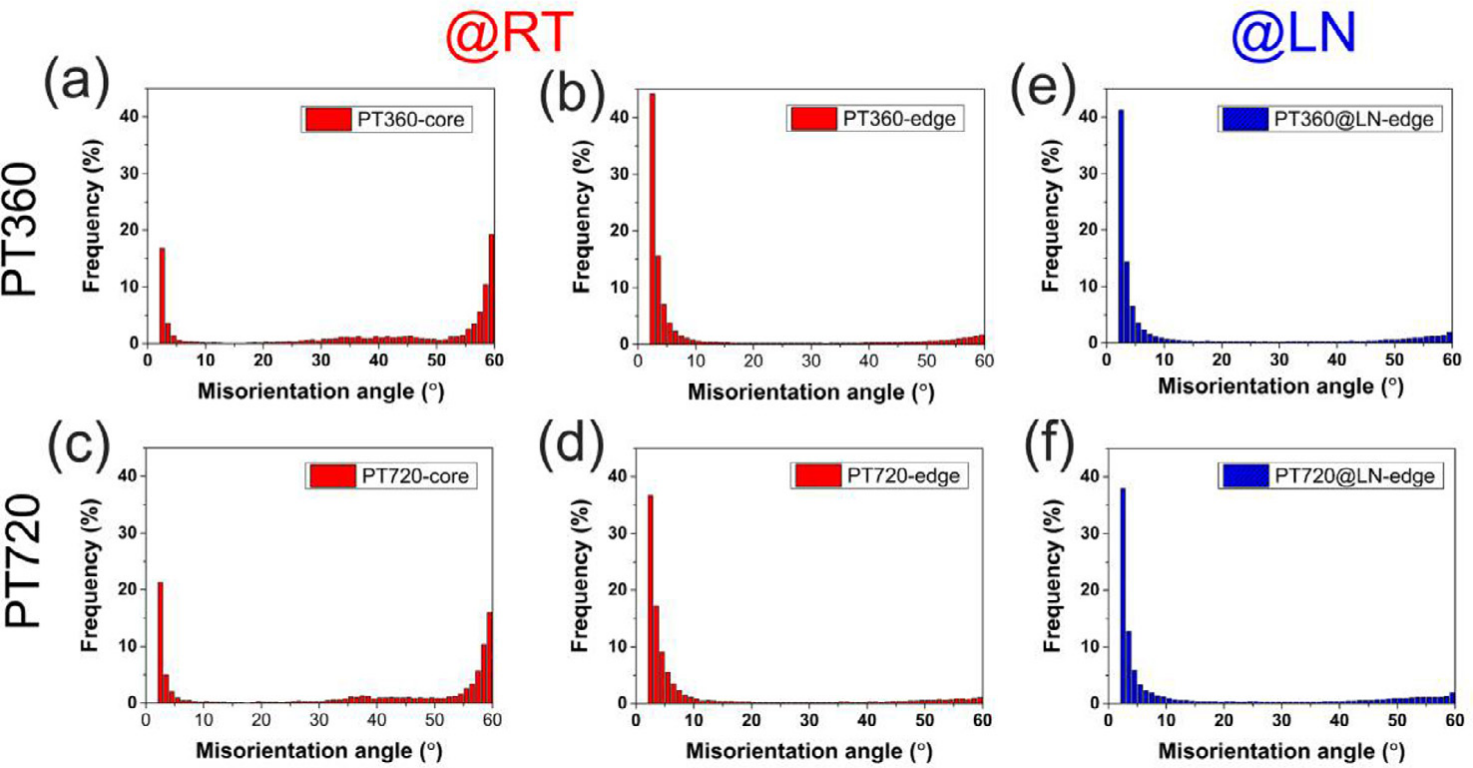
Misorientation angle distribution of various samples: (a) core of PT360; (b) surface of PT360; (c) core of PT720; (d) surface of PT720; (e) surface of PT360@LN; (f) surface of PT720@LN.

## 2 Experimental Design, Materials and Methods

Dog bone-shaped samples with a gauge length of 28 mm and a diameter of 4 mm were cut from the as-swaged (CrCoNi)_97_Al_1.5_Ti_1.5_ MEA rods. Unidirectional torsion was carried out on a free-end torsion machine at room temperature according to the model data in Fig. 4 (specific details of the experimental procedure were presented in Ref. [1]). The samples were twisted 360° and 720° at room temperature (denoted as PT360 and PT720, respectively) and 360° and 720° at liquid nitrogen temperature (denoted as PT360@LN), respectively.

### 2.1 X-ray diffraction analysis (XRD)

Phase identification of the surface of as-swaged and PT360@LN samples (multiple surface-layer pieces were spliced together, the area was about 64 cm^2^) was conducted on an X-ray diffraction instrument (MAXima XRD-6100, ShimadzuX-ray Diffractometer) with Cu Kα radiation, an angle range of 20–100°, and a step size of 0.02°. The phases were determined by using International Diffraction Data Center (ICDD) database, with the 2004 version of the powder diffraction file (PDF) database. The XRD curves of the two samples use the same abscissa, where the black line represents the as-swaged sample, and the red line represents the surface layer of the PT360@LN sample in Fig. 3.

### 2.2 SEM and EBSD analysis

The samples (as-swaged, core of PT360, surface of PT360, core of PT720, surface of PT720, surface of PT360@LN, surface of PT720@LN) were performed following conventional polishing procedures described in Ref. [1], and then were characterized with a scanning electron microscope (SEM, Zeiss Sigma HD) equipped with electron channeling contrast imaging (ECCI), electron diffraction spectrometer (EDS) and electron backscattered diffraction (EBSD, Oxford AZtech Max2) detectors. And the ECCI image and EDS images (see Fig. 1) can be obtained directly, while IPF maps, GB maps, KAM maps, misorientation angle distribution maps and pole figure can be calculated by the commercial software of HKL Channel5.

## Declaration of Competing Interest

None.

## Appendix Supplementary Materials

Image, application 1

## References

[1] N. Guo, Y.L. Zhao, S.T. Long, B. Song, J.J. Hu, B. Gan, L.J. Chai, S.F. Guo, Microstructure and mechanical properties of (CrCoNi)97Al1.5Ti1.5 medium entropy alloy twisted by free-end-torsion at room and cryogenic temperatures, Mater. Sci. Eng. A DOI: 10.1016/j.msea.2020.140101. 10.1016/j.dib.2020.106333 PMC7522488 33015260

[2] Humphreys F.J. (2004). Characterisation of fine-scale microstructures by electron backscatter diffraction (EBSD). Scr. Mater..

[3] Calcagnotto M, Ponge D., Demir E., Raabe D. (2010). Orientation gradients and geometrically necessary dislocations in ultrafine grained dual-phase steels studied by 2D and 3D EBSD. Mater. Sci. Eng. A.

